# Detecting “large language models fingerprint” for Japanese texts generated by six LLMs

**DOI:** 10.3389/frai.2026.1771115

**Published:** 2026-06-22

**Authors:** Wataru Zaitsu, Mingzhe Jin, Shunichi Ishihara, Satoru Tsuge, Mitsuyuki Inaba

**Affiliations:** 1 Faculty of Psychology, Mejiro University, Tokyo, Japan; 2 School of Artificial Intelligence, Jilin International Studies University, Jilin, China; 3 Institute of Interdisciplinary Research, Kyoto University of Advanced Science, Kyoto, Japan; 4 Speech and Language Laboratory, Australian National University, Canberra, ACT, Australia; 5 School of Informatics, Daido University, Aichi, Japan; 6 College of Policy Science, Ritsumeikan University, Kyoto, Japan

**Keywords:** Large language models (LLMs), stylometric analysis, function words, parts-of-speech, phrase patterns, UMAP, Random Forest, SHAP

## Abstract

**Background:**

Large language models (LLMs) have developed rapidly since the release of ChatGPT by OpenAI on November 30, 2022. This vogue comes to mind us “Can we distinguish LLMs-generated texts each other?” “What stylometric features are effective for differentiating LLMs such as fingerprints?” The purpose of this study was to distinguish the 300 Japanese texts (e.g., public comments) generated by six LLMs [ChatGPT (GPT5), Claude 3.5, Gemini, Microsoft Copilot, Llama 3.1, and Perplexity].

**Methods:**

To this end, we explored the effective stylometric features [e.g., function-word unigrams, part of speech (POS) bigrams, and phrase patterns] using the following analyses: (1) UMAP (Uniform Manifold Approximation and Projection) to visually explore distributional differences among LLMs, (2) Random Forest (RF) and XGBoost with leave-one-out cross-validation to differentiate LLMs, and (3) SHAP (SHapley Additive exPlanations) based on Random Forest to identify effective stylometric features.

**Results:**

First, UMAP demonstrated the separation of texts among the five LLMs except for Llama 3.1, which displayed substantial overlap with the other five LLMs. Second, RF achieved the highest performance across all stylometric features, with macro F1 scores exceeding 0.95 and reaching 1.00 for several LLMs. The detection performance of XGBoost was lower than that of RF, with the macro F1 scores ranging from 0.88 to 0.94. Finally, SHAP revealed LLM-specific patterns in function-word unigrams, POS bigrams, and phrase patterns.

**Conclusion:**

These findings indicate that Japanese public comments generated by the six LLMs can be accurately distinguished by focusing on the combination or patterns of stylometric features, suggesting LLM-specific linguistic fingerprints regardless of similarities in the underlying transformer architectures.

## 1 Introduction

Recently, various large language models (LLMs), such as ChatGPT, Google Gemini, and Perplexity, have been rapidly developed and widely adopted worldwide following the release of ChatGPT by OpenAI on November 30, 2022 (OpenAI, 2022). While this phenomenon has provided certain benefits, it has also generated emerging issues, including the spread of fake news in public policy, education, and marketing contexts. Several studies have reported that humans struggle to distinguish between artificial intelligence (AI)-generated and human-written texts based on appearance (Chein et al., 2024; Clark et al., 2021; Köbis and Mossink, 2021; Silva et al., 2024; Zaitsu et al., 2025a). However, recent findings suggest that machine-learning (ML) approaches can still discriminate between the two types of text in English (Berriche and Larabi-Marie-Sainte, 2024; Desaire et al., 2023; Kalmani et al., 2025; Przystalski et al., 2025), Chinese (Li and Zhang, 2025), and Japanese (Zaitsu and Jin, 2023; Zaitsu et al., 2024, 2025a). Desaire et al. (2023), using an Extreme Gradient Boosting (XGBoost) classifier, reported high performance (over 99%) in distinguishing between ChatGPT-3.5-generated and human-written academic papers. Berriche and Larabi-Marie-Sainte (2024) examined detection accuracy using both classical classifiers (k-nearest neighbors, decision tree, and Naïve Bayes) and ensemble classifiers (XGBoost and stacking), and demonstrated that classical classifiers achieved peak accuracies ranging from 96 to 98%. Regarding the Japanese language, a series of studies by Zaitsu and colleagues (Zaitsu and Jin, 2023; Zaitsu et al., 2024, 2025a) have consistently reported high detection accuracy. In particular, Zaitsu et al. (2025a) attempted to classify human-written texts and texts generated by seven different LLMs [ChatGPT (GPT-4o and o1), Claude 3.5, Gemini, Microsoft Copilot, Llama 3.1, and Perplexity] in a public comment corpus and achieved an accuracy of 99.8% using a Random Forest (RF) classifier. From the above findings, we can still distinguish between the LLM-generated text and human-written text. However, an important question remains: when considering only LLM-generated texts, can we meaningfully discriminate between them? This question is highly relevant to the forensic analysis of textual evidence. In forensic text comparisons, it is prudent to consider the possibility that the incriminating text was authored by a generative AI system. When a text is assessed as AI-generated, a technology capable of identifying the specific model or service used can provide useful investigative leads. Furthermore, the inability to differentiate a type of LLMs may arise from social, practical, and ethical issues, owing to the lack of clarity regarding the responsibility of each LLM developer. For example, such uncertainty can accelerate the spread of misinformation and disinformation, including propaganda, which politically motivates manipulation.

Current LLMs have been built upon underlined fundamental architecture known as the “transformer” model. The transformer, introduced by Vaswani et al. (2017) in “Attention Is All You Need,” triggered a paradigm shift in natural language processing. Modern LLMs are extensions or scaled-up implementations of this transformer architecture that incorporate improved training strategies, massive datasets, and optimized attention mechanisms. Therefore, although these LLMs share a common core architecture (a transformer), they have slightly different designs and training strategies. Are these subtle differences sufficient to distinguish the generated texts from one another? Najjar et al. (2025) attempted to differentiate essays generated by five LLMs (ChatGPT, Llama, Google Bard, Claude, and Perplexity) using RF, Recurrent Neural Networks (RNNs), and XGBoost. Their F1 results, including human-written essays, showed that the RF achieved the best score (93%), whereas the RNN models showed the lowest performance (74%). In particular, in the cases of ChatGPT and Llama using RF classifier, the accuracy reached 100%. Przystalski et al. (2025) also reported that successful LLMs (GPT-3.5/4, Llama 2/3, Orca, and Falcon) classification of human text, especially GPT-4, achieved the best accuracy (98%) on a balanced dataset. Limited to Japanese, Zaitsu et al. (2025a) utilized multidimensional scaling (MDS) to visually illustrate the distributional differences between LLM-generated and human-written texts. The findings showed that when focusing solely on LLM-generated texts, these distributions heavily overlapped and were not clearly separated in the MDS space, except for Llama 3.1. Therefore, Llama3.1 had specific patterns compared with the other LLMs. In a previous study, as stated before, when using the RF classifier, all LLMs (e.g., ChatGPT, Gemini, and Perplexity, etc.) were grouped under a single label “LLMs.” In other words, the analysis focused solely on distinguishing human-written texts from LLM-generated texts, and no attempt was made to differentiate between each LLM. To date, no study has examined the accuracy of distinguishing Japanese texts generated by LLMs.

Therefore, the purpose of this study was to analyze the distributional differences among several LLMs, attempt to classify their generated texts into each LLM type, examine detection performances, and explore effective stylometric features. For this purpose, we conducted the following three analyses: (1) Uniform Manifold Approximation and Projection (UMAP) to visually project differences in the dimensions among LLMs by focusing on several stylometric features, (2) RF and XGBoost to classify the texts into the class of each LLM following the framework of Najjar et al. (2025), and (3) SHapley Additive exPlanations (SHAP) based on RF to identify valid stylometric features. Using MDS, Zaitsu et al. (2025a) were unable to distinguish almost all LLMs except for Llama. Therefore, this study employed UMAP as an alternative method.

## 2 Materials and methods

### 2.1 Materials

We utilized 250 public comment texts generated by five LLMs (Claude 3.5, Gemini, Microsoft Copilot, Llama 3.1, and Perplexity) identical to those used in Zaitsu et al. (2025a). Only the ChatGPT texts (GPT-4o and o1) from the previous study were replaced with those generated by ChatGPT (GPT5), because more advanced models (GPT5) were released over time. Each LLM generated 50 texts using zero-shot prompts that specified only the role (e.g., general citizens or business people) and the title of the public comment. The roles and titles of the zero-shot prompts were consistent across all the LLMs. For example, first text of GPT5 and Gemini constitute one pair with the same content (e.g., public comment for “Basic Policy on Support for the Independence of Homeless Persons”), whereas second texts of GPT5 and Gemini constitute another pair with different but internally consistent contents (e.g., public comment for “Guidelines for Telework Security”). A total of 50 texts were arranged in content-matched pairs. A plausible misuse scenario for generative AI in the context of public commentary is a deliberate attempt to influence policy decision-making. In such cases, the malicious actor is likely to be a user of generative AI, and therefore relies on default settings with a minimal prompt. This rationale supports the use of a simple zero-shot prompt in realistic threat scenarios. In addition, we generated texts using the default hyperparameter settings of each LLM (e.g., temperature and top-p) via their standard user interfaces (i.e., without API-based control).

The mean (standard deviation) for the characters of texts generated by each LLM was 766.2 (91.6) for GPT5, 710.8 (120.6) for Claude3.5, 912.8 (140.9) for Gemini, 823.8 (91.9) for Microsoft Copilot, 919.7 (179.0) for Llama3.1, and 1136.9 (215.7) for Perplexity.

### 2.2 Japanese stylometric features

This study uses three Japanese stylometric features similar to those used by Zaitsu et al. (2025a): (1) function-word unigrams, (2) part of speech (POS) bigrams, and (3) phrase patterns. These stylometric features are considered to be effective for authorship analysis (Jin, 2004, 2013; Zaitsu and Jin, 2018) and generative AI detection for differentiating humans (Zaitsu and Jin, 2023; Zaitsu et al., 2024, 2025a). However, Zaitsu et al. (2025a) reported that these three Japanese stylometric features were not effective in differentiating the texts generated by each LLM. Therefore, this study conducted different analysis methods using the same stylometric features and compared them with those of this previous study.

#### 2.2.1 Function-word unigrams

Function words have no semantic content but serve grammatical roles in a sentence (e.g., “は” and “を” as postpositional particles, “です” and “だ” as auxiliary verb, and “なお” and “しかし” as conjunction in Japanese). Compared to nouns, these features are relatively independent of topical content; in particular, Japanese postpositional particles have been verified to be comparatively robust against topic variations (Zaitsu et al., 2025b). In addition, we treated punctuation, such as Japanese commas, and periods, as function words.

#### 2.2.2 POS bigrams

These features, defined *N* = 2 in N-grams based on POS, are frequently used in authorship attribution and generated AI detection because of their effectiveness. In case of Japanese sentence “私は本を読む” (“I read this book”), we can segment this sentence and attach POS-tag by morphological analysis as follows: “私 (noun),” “は (particle),” “本 (noun),” “を (particle),” and “読む (verb).” Additionally, the POS bigrams correspond to combinations such as “noun + particle,” “particle + noun,” and “particle + verb.” These POS-level co-occurrence patterns are likely to capture grammatical sequencing, rather than lexical content. Therefore, these features are also considered robust against meanings.

#### 2.2.3 Phrase patterns

This approach, originally proposed by Jin (2013), focuses on the structural configuration within each phrase by combining function words (e.g., “は,” “だ,” and “そして”) with content words transformed into their corresponding POS categories (e.g., “noun,” “verb,” and “adjective”). In this study, phrase patterns were extracted using the following multi-step preprocessing pipeline. First, each sentence was segmented into phrase-level units using syntactic analysis. Second, we conduct a morphological analysis to decompose the sentences within each phrase into morpheme-level units and assign POS-tags. Finally, content words were masked with their POS labels (e.g., “noun” or “verb” etc.), and function words were retained in their original form to avoid the effects of topic-specific vocabulary as much as possible. As a result of these procedures, the example sentence “私は本を読む” is transformed into “noun + は/ noun + を/ verb,” where the slash (/) is boundary between two phrases.

#### 2.2.4 Integrated features

This study integrated the above three features (function-word unigrams, POS bigrams, and phrase patterns) as “integrated features” for examining whether integrating stylometric features improve detection performances (incremental validity).

In analyzing these stylometric features, we computed how often each feature appeared in the texts of each LLM and normalized these count values by dividing them by the total number of stylometric features observed in the same text (relative frequencies) to avoid bias stemming from differences in text length. Furthermore, to analyze the above stylometric features, we conducted a morphological analysis to annotate Word-level POS using the Japanese POS tagger MeCab (ver. 0.996, Kudo et al., 2004). Additionally, a syntactic analysis for divided sentences into phrase-level units was conducted using the Japanese parser CaboCha (ver. 0.69, Kudo and Matsumoto, 2002).

### 2.3 Analysis methods and procedure

This study adopts the following analyses: (1) UMAP, (2) RF, and XGBoost, and (3) SHAP based on RF.

#### 2.3.1 UMAP

It is a dimensionality-reduction algorithm that projects high-dimensional data into two or three dimensions, similar to principal component analysis (PCA) or t-SNE. Notably, UMAP emphasizes the local neighborhood structure, preserves nearest-neighbor relationships, and maintains the integrity of small clusters. This property enables the intuitive visualization of group-level relationships. Compared with other dimensionality-reduction algorithms (e.g., MDS, PCA, and t-SNE), UMAP is advantageous because of its superior ability to explore clustering structures and preserve sample-level neighborhood information (Yang et al., 2021). In this study, we utilized the *umap* function of the **umap** package (ver. 0.2.10.0) in the R language (ver. 4.5.0).

#### 2.3.2 Random forest

RF is an ensemble learning approach based on a large number of decision tree trainings using bootstrap samples of data (Breiman, 2001). The decision algorithm is a characteristic of a majority vote by multiple weak decision tree classifiers. RF is considered an effective method for authorship analysis and AI detection. For the RF analysis, we used the *randomForest* function of the **randomForest** package (ver. 4.7-1.2) in R language and set the number of trees to 1,000 (ntree) to ensure stable and reliable classification performance. The other RF hyperparameters (e.g., mtry, nodesize, and replace) were maintained at their default settings in the **randomForest** package.

#### 2.3.3 XGBoost

This approach is a highly efficient gradient-boosting framework similar to RF in terms of ensemble learning and weak classifiers based on multiple decision trees. However, the decision trees of the RF are used in parallel, whereas XGBoost treats the decision trees in series. In other words, XGBoost builds trees sequentially using gradient boosting, in which each new tree is trained to correct the errors of the previous ones. XGBoost was learned using the *xgb.train* function of the **xgboost** package (ver. 3.1.3.1) in R language. The XGBoost hyperparameters were set as follows: the maximum tree depth was set to 6 (max_depth = 6) and the learning rate to 0.1 (eta = 0.1), with 200 boosting iterations (nrounds = 200). The other parameters were maintained at default values.

In analyzing RF and XGBoost, we conducted Leave-One-Out Cross-Validation (LOOCV) to examine the generalization performance. LOOCV is a rigorous evaluation procedure in which the model is trained on all samples except one and the held-out sample is used for testing. This one-set process was repeated for each sample.

#### 2.3.4 SHAP

This algorithm is based on Shapley values that explain how features (stylometric features) contribute to machine-learning predictions or classifications (Lundberg and Lee, 2017). Shapley values stem from game theory and quantify the extent to which each feature enhances or deteriorates the detection performance of classifier prediction or classification compared with a baseline. We adopted SHAP because it provides feature importance scores along with the direction of influence (positive or negative), and can calculate the mean (|SHAP|) to examine the magnitude and patterns of the influences.

### 2.4 Detection performance metrics

In this study, each metric (accuracy, recall, precision, and macro F1) was estimated from confusion matrix. For example of Table 1, for ChatGPT(GPT5), we calculated Equations 1–7.


$$\begin{array}{l} Accuracy=\frac{a_{1}+b_{2}+c_{3}+d_{4}+e_{5}+f_{6}}{all\;texts\;\left(N=\;300\right)} \end{array}$$ (1)

$$\begin{array}{l} Recall\;\left(GPT5\right)=\frac{a_{1}}{\overset{6}{\underset{j=1}{\sum }}a_{j}} \end{array}$$ (2)

$$\begin{array}{l} Precision\;\left(GPT5\right)=\frac{a_{1}}{a_{1}+b_{1}+c_{1}+d_{1}+e_{1}+f_{1}} \end{array}$$ (3)

$$\begin{array}{l} F1\;\left(GPT5\right)=\frac{2\times Recall\left(GPT5\right)\times Precision\left(GPT5\right)}{Recall\left(GPT5\right)+Precision\left(GPT5\right)} \end{array}$$ (4)

Moreover, equations of macro average for all six LLMs were as follows.


$$\begin{array}{l} Macro\;average\;for\;recall=\frac{Recall\;for\;all\;six\;LLMs\;}{6} \end{array}$$ (5)

$$\begin{array}{l} Macro\;average\;for\;precision=\frac{Precision\;for\;all\;six\;LLMs\;}{6} \end{array}$$ (6)

$$\begin{array}{l} Macro\;average\;for\;F1=\frac{F1\;for\;all\;six\;LLMs\;}{6} \end{array}$$ (7)

**Table 1:** Example of confusion matrix.

True class	Classified class					
	ChatGPT(GPT5)	Claude3.5	Microsoft Copilot	Gemini	Llama3.1	Perplexity
ChatGPT(GPT5)	*a*_1_	*a*_2_	*a*_3_	*a*_4_	*a*_5_	*a*_6_
Claude3.5	*b*_1_	*b*_2_	*b*_3_	*b*_4_	*b*_5_	*b*_6_
Microsoft Copilot	*c*_1_	*c*_2_	*c*_3_	*c*_4_	*c*_5_	*c*_6_
Gemini	*d*_1_	*d*_2_	*d*_3_	*d*_4_	*d*_5_	*d*_6_
Llama3.1	*e*_1_	*e*_2_	*e*_3_	*e*_4_	*e*_5_	*e*_6_
Perplexity	*f*_1_	*f*_2_	*f*_3_	*f*_4_	*f*_5_	*f*_6_

## 3 Results

### 3.1 UMAP projection of Japanese texts generated by six LLMs

**Figure 1:**
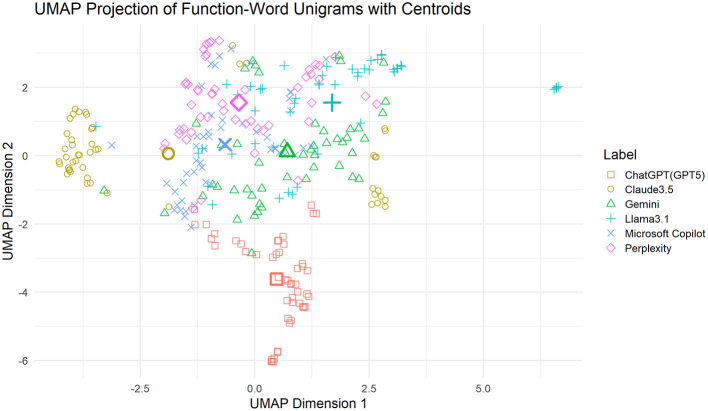
UMAP projection for function-word unigrams in six LLMs. Each enlarged marker denotes the centroid (median UMAP coordinates) of texts within each LLM, highlighting group-level stylometric tendencies.

**Figure 2:**
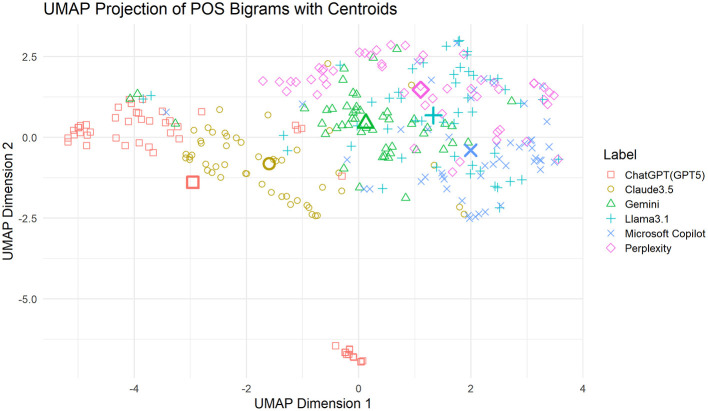
UMAP projection for POS bigrams in six LLMs.

**Figure 3:**
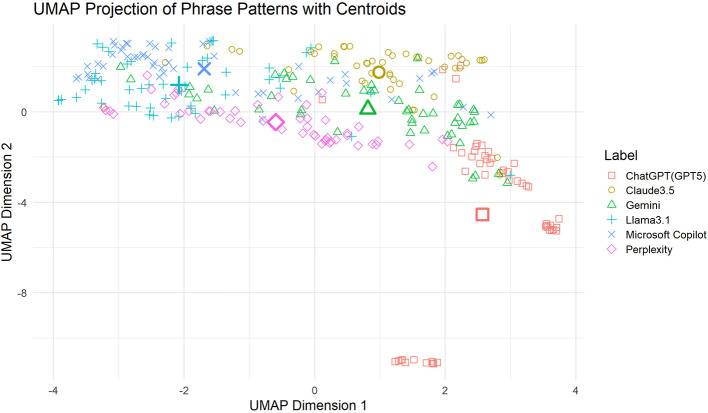
UMAP projection for phrase patterns in six LLMs.

**Figure 4:**
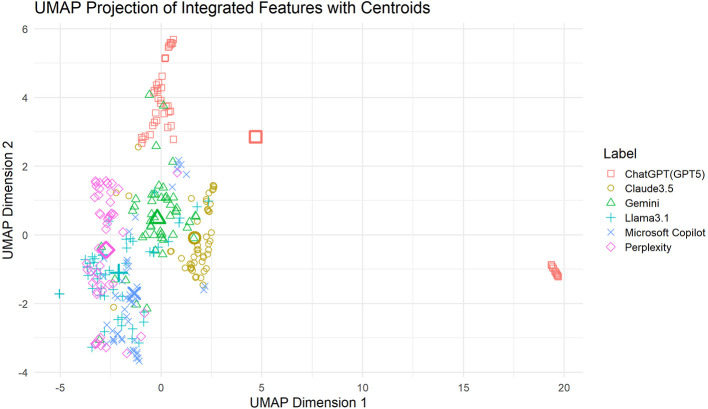
UMAP projection for integrated features in six LLMs.

The results of UMAP results are shown in the following figures: Figure 1 (function-word unigrams), Figure 2 (POS bigrams), Figure 3 (phrase patterns), and Figure 4 (integrated features). As shown in Figure 1, GPT5 and Claude3.5 were visually separated from the texts of the other LLMs. Additionally, focusing on only Claude3.5, the two distinct text clusters were far apart from each other in the UMAP space. The text clusters of both Gemini and Perplexity overlapped with those of Llama3.1 and Microsoft Copilot. However, except for Gemini and perplexity, Llama3.1 and Microsoft Copilot were likely to separate from each other. Figure 2 for POS bigrams also shows that almost all of the texts of GPT5 and Claude3.5 were different distributions from the texts of the other LLMs. In particular, some of the texts of GPT5 were quite differently located on the dimension compared to the other texts. Additionally, while Llama3.1 texts overlapped with Gemini, Perplexity, and Microsoft Copilot texts, excluding Llama3.1 texts. These three LLMs tended to separate from each other. Figure 3 shows that the texts of Llama3.1 and Gemini overlapped with the texts of the other LLMs. However, focusing on the other four LLMs (GPT5, Perplexity, Claude3.5, and Microsoft Copilot), these texts showed little overlap. Finally, the integrated features (Figure 4) also showed that almost all of the Llama3.1 texts overlapped with the other LLMs' texts. However, except for Llama3.1, five LLMs did not overlap. Therefore, only Llama3.1 texts were likely to overlap consistently and had difficulty visually distinguishing as opposed to Zaitsu et al. (2025a). In addition, Gemini was also considered a relatively indistinguishable LLM.

### 3.2 Performance metrics of the RF

The function-word unigrams listed in Table 2 show that ChatGPT(GPT5) achieved 100% on F1. Even for the lowest-performing model, Microsoft Copilot achieved a 94.8% on macro F1. The mean averages of each metric were as follows: recall (0.961), precision (0.960), and F1 (0.960). The POS bigrams listed in Table 2 demonstrated higher performance levels; Among these results, Llama3.1 showed 100% F1. For the same function-word unigrams, the detection performance of Microsoft Copilot had the lowest F1. The macro averages of each metric were as follows: recall (0.968), precision (0.972), and F1 (0.970). According to the phrase patterns, two LLMs (Llama3.1 and perplexity) achieved the best performance (100% F1). In contrast to function-word unigrams and POS bigrams, Claude3.5 had the lowest value for F1. The macro metrics were recall (0.956), precision (0.975), and F1 (0.965). The results of the integrated features also demonstrated 100% for F1 on Llama3.1 and Perplexity. The macro recall and precision were calculated as 0.968 and 0.972, respectively. Although we integrated three stylometric features to enhance performance, there was not much difference in the macro F1 between the POS bigrams (0.970) and the integrated features (0.970).

**Table 2:** Accuracy obtained in focusing on each feature and analyzing the texts using RF.

Stylometric features and LLMs	Accuracy	Recall	Precision	F1
Function-words unigrams				
ChatGPT(GPT5)	0.967	1.000	1.000	1.000
Claude3.5		0.980	0.925	0.951
Microsoft Copilot		0.920	0.979	0.948
Gemini		0.920	1.000	0.958
Llama3.1		1.000	0.962	0.980
Perplexity		0.980	0.942	0.961
POS bigrams				
ChatGPT(GPT5)	0.973	1.000	0.980	0.990
Claude3.5		0.960	0.941	0.950
Microsoft Copilot		0.920	0.979	0.948
Gemini		0.960	0.960	0.960
Llama3.1		1.000	1.000	1.000
Perplexity		1.000	0.980	0.990
Phrase patterns				
ChatGPT(GPT5)	0.963	1.000	0.909	0.952
Claude3.5		0.920	0.939	0.929
Microsoft Copilot		0.920	0.979	0.948
Gemini		0.940	0.959	0.949
Llama3.1		1.000	1.000	1.000
Perplexity		1.000	1.000	1.000
Integrated features				
ChatGPT(GPT5)	0.973	1.000	0.980	0.990
Claude3.5		0.960	0.923	0.941
Microsoft Copilot		0.920	0.979	0.948
Gemini		0.960	0.960	0.960
Llama3.1		1.000	1.000	1.000
Perplexity		1.000	1.000	1.000

### 3.3 Performance metrics of the XGBoost

As listed in Table 3, the function-word unigrams suggested that the texts generated by Llama3.1 were the most readily distinguishable, whereas the macro F1 of Microsoft Copilot dropped below 90%. In addition, the macro F1 (0.938) of XGBoost was lower than that in the RF analysis (0.960). The other average metrics calculated were macro recall (0.936) and macro precision (0.940). The detection performance for POS bigrams deteriorated markedly across all metrics. In particular, the F1 score for the Gemini dropped below 0.800. The macro metrics also deteriorated as follows: macro recall (0.876), macro precision (0.880), and macro F1 (0.876). Half of the LLMs focusing on phrase patterns did not reach 90% on F1 scores. Overall, the scores obtained using XGBoost were lower than those obtained using RF. The macro metrics were recall (0.908), precision (0.919), and F1 (0.913). Finally, we did not observe the incremental validity of the integrated features because they did not show the best macro F1 score compared with each single feature, as follows: 0.920 (macro recall), 0.922 (macro precision), and 0.920 (macro F1).

**Table 3:** Accuracy obtained in focusing on each feature and analyzing the texts using XGBoost.

Stylometric features and LLMs	Accuracy	Recall	Precision	F1
Function-words unigrams				
ChatGPT(GPT5)	0.943	0.980	0.961	0.970
Claude3.5		0.900	0.918	0.909
Microsoft Copilot		0.880	0.898	0.889
Gemini		0.940	0.959	0.949
Llama3.1		0.980	1.000	0.990
Perplexity		0.980	0.925	0.951
POS bigrams				
ChatGPT(GPT5)	0.887	0.940	0.922	0.931
Claude3.5		0.780	0.867	0.821
Microsoft Copilot		0.920	0.807	0.860
Gemini		0.760	0.826	0.792
Llama3.1		1.000	0.962	0.980
Perplexity		0.920	0.939	0.929
Phrase patterns				
ChatGPT(GPT5)	0.903	0.880	0.830	0.854
Claude3.5		0.880	0.880	0.880
Microsoft Copilot		0.940	0.940	0.940
Gemini		0.840	0.913	0.875
Llama3.1		0.940	0.940	0.940
Perplexity		0.940	0.922	0.931
Integrated features				
ChatGPT(GPT5)	0.923	0.940	0.940	0.940
Claude3.5		0.920	0.836	0.876
Microsoft Copilot		0.860	0.915	0.887
Gemini		0.900	0.918	0.909
Llama3.1		1.000	1.000	1.000
Perplexity		0.920	0.939	0.929

### 3.4 SHAP-based feature importance

**Figure 5:**
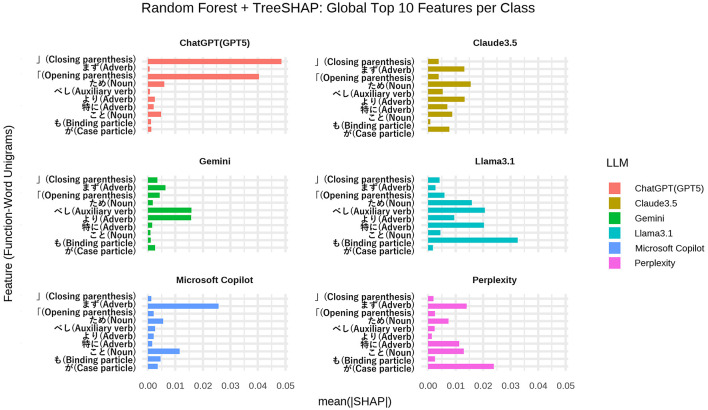
Global Top 10 SHAP based on function-word unigrams on each LLM using Random Forest.

**Figure 6:**
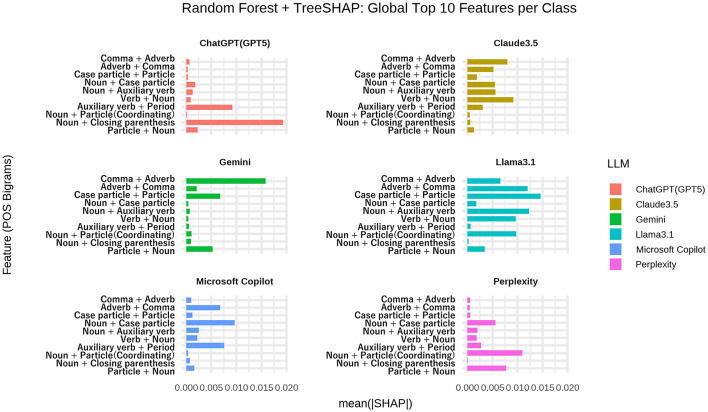
Global Top 10 SHAP based on POS bigrams on each LLM using Random Forest.

**Figure 7:**
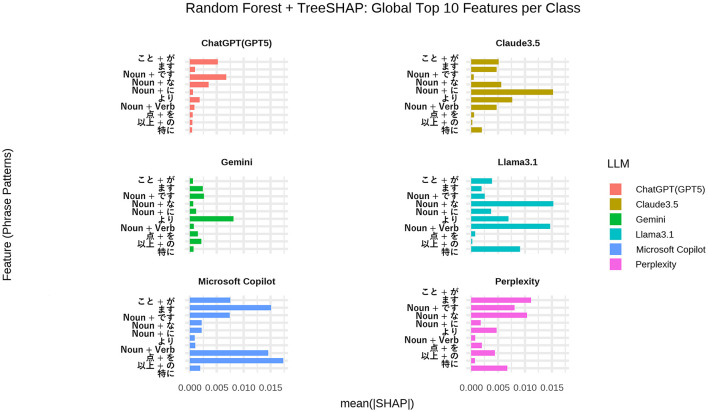
Global Top 10 SHAP based on phrase patterns on each LLM using Random Forest.

**Figure 8:**
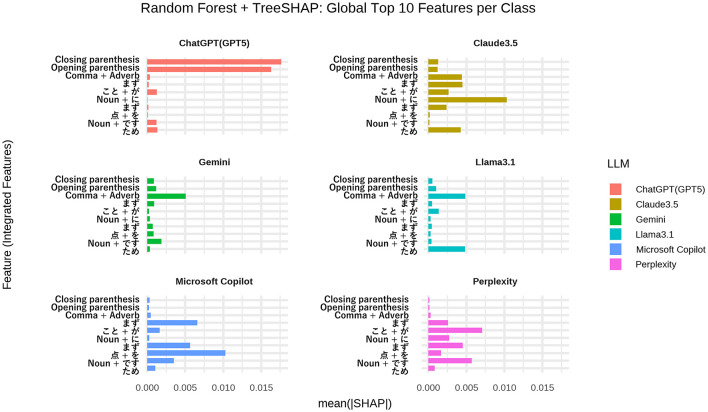
Global Top 10 SHAP based on integrated features on each LLM using Random Forest.

We calculated the SHAP scores of each stylometric feature to interpret which stylometric features the RF classifier focused on across each LLM, identified the 10 features with the largest global importance (the highest mean |SHAP| averaged over all LLMs), and represented their class-wise contributions (Figures 5–8). As shown in Figure 5 (function-word unigrams), the SHAP profiles revealed model-specific patterns as “fingerprints.” The three LLMs [ChatGPT(GPT5), Gemini, and Microsoft Copilot] were characterized by two highly dominant features, whereas the other LLMs, Claude3.5, Llama3.1, and Perplexity, showed a more distributed pattern with several function words that contributed moderately to the classification. For more details, almost all of the LLMs generally relied on parenthesis symbol, adverb (e.g., “まず (initially),” “特に (especially),” “より (more)”), particles (e.g., “も” and “が”), and auxiliary verb (e.g., “べし”). Figure 6 shows that the importance patterns of the POS bigrams varied across LLMs. This can be classified into two types: the first type (GPT5, Gemini, Microsoft Copilot, and Perplexity) has two or three dominant POS bigrams (e.g., “noun + parentheses,” “commas + adverb,” or “noun + particle”), and the other type (Claude3.5 and Llama3.1) shows multiple combinations of POS bigrams. The phase patterns shown in Figure 7 exhibit several distinctive profiles. Among the LLMs, Gemini is characteristics as representing only word “より.” The other LLMs were distinguishable from each other by focusing on combinations of several features within each phrase (e.g., “noun + に,” “こと + が,” “いくつ + か + の”). As shown in the integrated feature, the SHAP analysis revealed that high-level patterns (POS bigrams and phrase patterns) were likely to distinguish almost all LLMs rather than focusing on single features (function-word unigrams). Additionally, Figure 8 demonstrates that several LLMs (GPT5, Gemini, and Llama3.1) showed one or two distinctive feature (e.g., “parenthesis symbol,” “commas + adverb,” and “ため + noun”), whereas the other LLMs (Claude3.5, Microsoft Copilot, and perplexity) need combine multiple features. As mentioned above, the SHAP results suggest that LLMs differ in the stylometric features that contribute to their classification, with some models showing few dominant features and others exhibiting more distributed patterns.

## 4 Discussion

First, we aimed to distinguish six LLMs visually using UMAP, focusing on three Japanese stylometric features (function-word unigrams, POS bigrams, and phrase patterns). Consequently, Llama3.1 texts were indistinguishable across all stylometric features in the analysis of UMAP because they generally overlapped with the other LLMs. In contrast, the other LLMs (GPT5, Claude3.5, Microsoft Copilot, and Perplexity), except for Llama3.1, were likely to demonstrate different positions on the UMAP dimensions. In contrast to these results, a study (Zaitsu et al., 2025a) using MDS revealed that only Llama3.1 was distinguishable because it was clearly separated from the other LLMs. Therefore, using multiple visual exploratory techniques, including MDS, UMAP, and t-SNE, to arrange texts into different dimensions may provide various perspectives, thereby enhancing detection performance. In addition, the observed superiority of RF may be attributed to the interaction between the algorithmic properties and structural characteristics of the texts (i.e., high dimensionality, sparsity, noise, and limited sample size). Text representations, such as N-grams, typically result in high-dimensional and sparse feature spaces that are weakly informative or noisy. Under these conditions, RF exhibits robustness owing to its bagging mechanism, whereas boosting methods such as XGBoost iteratively focus on hard-to-classify instances. Although effective in many settings, this sequential emphasis can lead to overfitting or instability when the data are sparse and noisy. Given the small sample size, RF tended to maintain a stable performance, as opposed to XGBoost.

Second, this study revealed that the detection performance of RF is superior to that of XGBoost. This finding is consistent with that of Najjar et al. (2025) who targeted English texts. That is, although the lowest performance metrics of XGBoost fell below 70%, those of RF did not decrease below 90%. Regarding the XGBoost hyperparameters, as previously stated, we did not conduct grid search to adjust for the best performance. Therefore, grid search may improve detection performance. Furthermore, in the case of ChatGPT(GPT5), Llama3.1, and Perplexity, the F1 of RF reached 100% using stylometric features. However, this study discovered no incremental validity in combining the three stylometric feature sets, as the integrated features did not further improve accuracy. This may reflect a ceiling effect, that is, a situation in which a large proportion of observations achieve the maximum performance level (100%), leaving little room for improved accuracy.

Consequently, UMAP cannot differentiate Llama3.1 from other LLMs, but RF can classify the texts generated by Llama3.1 into the class of Llama3.1. This discrepancy between UMAP and RF results from their fundamentally different objectives and computational properties. UMAP visually reduces high-dimensional data to two-dimensional space and discards subtle stylometric cues. In contrast, RF classifiers learn from the full high-dimensional feature space and can capture complex nonlinear boundaries. The SHAP results (Figures 5–8) show that the identification of Llama3.1 relies on subtle and distributed stylometric features (e.g., “comma + adverb” and “ため”), which are not preserved in low-dimensional projections. Therefore, regardless of whether the texts of Llama3.1 overlapped with the other LLM texts and appeared indistinguishable in a two-dimensional space, RF can detect subtle cues of Llama3.1 and can successfully capture Llama3. Furthermore, UMAP revealed that the texts generated by Claude 3.5 (Figure 1) and ChatGPT (GPT5) (Figure 4) formed two distinct clusters, suggesting intra-model variability. This finding indicates that even within a single LLM, stylistic outputs may not be homogeneous but may instead reflect multiple latent generation patterns, potentially influenced by subtle variations in internal sampling processes or response strategies. Taken together, these findings suggest that the “LLM fingerprint” identified in this study should be interpreted as preliminary and context-dependent rather than fixed or universal.

Finally, SHAP based on RF clarified that LLMs exhibited distinct patterns of stylometric features like “fingerprints” regardless of their shared transformer-based architectures. It is considered that the differences in stylometric feature patterns may be caused by the following factors: (1) variations in the pre-training corpora, (2) differences in tokenization schemes that determine how Japanese text is segmented and encoded during training, and (3) differences in Reinforcement Learning from Human Feedback (RLHF) and generation policies of companies, both of which shape stylistic preferences and impose constraints on tone, safety, and discourse structure.

Several limitations must be acknowledged. First, the dataset consisted of a small number of texts (*N* = 300, 50 per LLM), which may limit the generalizability of the findings. Although we conducted LOOCV to maximize the use of available data, the near-perfect classification performance observed under some conditions (e.g., F1 = 1.00) may partly reflect the characteristics of this controlled dataset, rather than the fully robust and generalizable stylometric features of each LLM. Therefore, future studies should validate these findings by using larger and more diverse corpora, including different text genres, prompting strategies, and languages, to examine the robustness and generalizability of LLM-specific stylometric patterns. Second, the dataset consisted solely of public comment texts, which may limit the generalizability of the results to other genres, such as academic writing, conversational dialogue, or social media posts. Third, this study focused only on Japanese texts. Given that Japanese differs from other languages, including writing systems (kanji, hiragana, and katakana) and the absence of explicit word boundaries, the findings may not be generalizable to other languages with different linguistic structures and stylometric properties.

This study concluded that texts generated by multiple LLMs can be distinguished by focusing on stylometric features. These results suggest that the responsibility of LLM developers can be clarified in issues related to opinion manipulation (e.g., election interference, propaganda, and the manipulation of public discourse). However, the patterns of stylometric features of texts generated by each LLM may change as new LLMs are developed and are not based on the transformer architecture. Therefore, similar studies should be conducted in the future.

## 5 Future studies

We propose that future studies need to verify “LLMs fingerprint” under different factors as follows: (1) Different LLMs as now existing (e.g., Mistral AI, Qwen, and Gemma) or new emerging LLMs, (2) Different genres texts instead of public comments, (3) Larger and more diverse dataset of texts, (4) One- or few-shot prompting during generating texts as opposed to this study generated by zero-shot prompting, and (5) Multiple languages and evaluating their robustness beyond Japanese.

## Data Availability

The original contributions presented in the study are included in the article/Supplementary material, further inquiries can be directed to the corresponding author.
