# Correction: Detecting “large language models fingerprint” for Japanese texts generated by six LLMs

**DOI:** 10.3389/frai.2026.1916608

**Published:** 2026-07-15

**Authors:** Wataru Zaitsu, Mingzhe Jin, Shunichi Ishihara, Satoru Tsuge, Mitsuyuki Inaba

**Affiliations:** 1 Faculty of Psychology, Mejiro University, Tokyo, Japan; 2 School of Artificial Intelligence, Jilin International Studies University, Jilin, China; 3 Institute of Interdisciplinary Research, Kyoto University of Advanced Science, Kyoto, Japan; 4 Speech and Language Laboratory, Australian National University, Canberra, ACT, Australia; 5 School of Informatics, Daido University, Aichi, Japan; 6 College of Policy Science, Ritsumeikan University, Kyoto, Japan

**Keywords:** large language models (LLMs), stylometric analysis, function words, parts-of-speech, phrase patterns, UMAP, Random Forest, SHAP

Affiliation “School of Artificial Intelligence, Jilin International Studies University, Jilin, China” was added as a present address instead of a main affiliation. This affiliation has now been added to the main affiliation list for author(s) [Mingzhe Jin].

The original version of this article has been updated.

